# Potentially Zoonotic Enteric Infections in Gorillas and Chimpanzees, Cameroon and Tanzania

**DOI:** 10.3201/eid3003.230318

**Published:** 2024-03

**Authors:** Emily K. Strahan, Jacob Witherbee, Richard Bergl, Elizabeth V. Lonsdorf, Dismas Mwacha, Deus Mjungu, Mimi Arandjelovic, Romanus Ikfuingei, Karen Terio, Dominic A. Travis, Thomas R. Gillespie

**Affiliations:** Emory University, Atlanta, Georgia, USA (E.K. Strahan, E.V. Lonsdorf, T.R. Gillespie);; Centers for Disease Control and Prevention, Atlanta (J. Witherbee);; North Carolina Zoo, Asheboro, North Carolina, USA (R. Bergl);; Jane Goodall Institute Tanzania, Kigoma, Tanzania (D. Mwacha, D. Mjungu);; Max Planck Institute for Evolutionary Anthropology, Leipzig, Germany (M. Arandjelovic);; iDiv, German Centre for Integrative Biodiversity Research Halle-Jena-Leipzig, Leipzig (M. Arandjelovic);; Wildlife Conservation Society Takamanda-Mone Landscape Project, Limbe, Cameroon (R. Ikfuingei);; University of Illinois, Urbana, Illinois, USA (K. Terio);; University of Minnesota, Minneapolis, Minnesota, USA (D.A. Travis)

**Keywords:** enteric infections, zoonoses, disease reservoirs, One Health, primates, primate diseases, Cameroon, Tanzania, bacteria, viruses, parasites

## Abstract

Despite zoonotic potential, data are lacking on enteric infection diversity in wild apes. We employed a novel molecular diagnostic platform to detect enteric infections in wild chimpanzees and gorillas. Prevalent *Cryptosporidium parvum*, adenovirus, and diarrheagenic *Escherichia coli* across divergent sites and species demonstrates potential widespread circulation among apes in Africa.

The close phylogenetic relationship between humans and great apes results in similarities in infection susceptibility and a high potential for pathogen exchange (*1*,*2*). Despite this zoonotic potential, previous studies of wild great apes have targeted specific infections (*3*,*4*), failing to establish baselines of the diversity of potentially zoonotic infections in these species. To improve our understanding of which enteric infections great apes are exposed to, we examined biobanked fecal samples from 2 biogeographically and phylogenetically divergent wild great ape species in Africa for an array of viral, parasitic, and bacterial enteric targets using a novel real-time PCR diagnostic platform. 

## The Study

During December 2011–January 2012, a total of 58 fecal samples from critically endangered Cross River gorillas (*Gorilla gorilla diehli*) were noninvasively collected from nest sites and along trails from 2 sites in Cameroon, as detailed in Arandjelovic et al. (*5*). Sampled gorillas experienced infrequent overlap with humans engaged in research or extraction of nontimber forest products (*5*). Fecal DNA extract and microsatellite genotyping identified individual gorilla sample donors, confirming repeated sampling of 18 gorillas: 10 from Kagwene Gorilla Sanctuary (≈50% of population) and 8 from Mone River Forest Reserve (≈35%–40% of population) (*5*). Given that serial sampling can increase chances that an individual tests positive for a target (*6*), only the first sample collected from each gorilla was screened.

During September 2016–February 2018, fecal samples were noninvasively collected from each of 56 individually recognized endangered eastern chimpanzees (*Pan troglodytes schweinfurthii*) (≈50% of population) from Gombe National Park, Tanzania, as detailed in Wroblewski et al. (*7*). Sampled chimpanzees experienced daily overlap with humans engaged in research and tourism following best practices to reduce the risk for pathogen exchange (*2*) and experienced infrequent overlap with humans when consuming crops at the boundary of the protected area (*8*). Fecal DNA extract and microsatellite genotyping were used to identify individual chimpanzee sample donors (*7*).

For all apes sampled, fresh fecal samples were preserved upon collection in Ambion RNAlater (MilliporeSigma, https://www.sigmaaldrich.com) and stored at −20°C until shipping to the United States, where they were stored at −80°C until thawed for extraction. In December 2019, we used the TaqMan Array Card (ThermoFisher Scientific, https://www.thermofisher.com), a novel real-time PCR testing platform, to screen ape fecal samples for 39 unique enteric pathogen targets (Appendix). Targets were pathogen-specific genes associated with either virulence or biology (i.e., specific outer membrane protein genes or housekeeping genes). As detailed in Diaz et al. (*8*), we extracted DNA and RNA from each fecal specimen using a Roche MagNA Pure Compact magnetic bead Total Nucleic Acid Kit (Roche, https://www.roche.com). For preprocessing, we incubated sample, lysis buffer, and proteinase (56°C, 15 minutes) before 2 cycles on Precellys bead-beater (Bertin Technologies, https://www.bertin-technologies.com) at 5,000 rpm for 60 seconds. We assayed extracts using the Applied Biosystems ViiA7 Real-Time PCR system (ThermoFisher Scientific) with the following cycling conditions: 45°C for 10 minutes, 94°C for 10 minutes, 45 cycles of 94°C for 30 seconds, and 60°C for 60 seconds (*8*). For validation, we spiked fecal samples with known DNA/RNA concentrations. We evaluated sensitivity with spiked dilution series and specificity through BLAST (https://blast.ncbi.nlm.nih.gov/Blast.cgi) then isolate panel representing targeted organisms. We evaluated exclusivity using nucleic acid from closely related species.

Analyses confirmed presence of nucleic acids of >1 enteric pathogen target in 15 (83%) of the 18 gorillas and 39 (70%) of the 56 chimpanzees. We detected 7 pathogen targets among gorillas (Table 1) and 13 among chimpanzees (Table 2). Adenovirus and *Cryptosporidium parvum* were the most common pathogen targets detected in both gorillas and chimpanzees, occurring in 33% (95% CI 10%–57%) (adenovirus) and 39% (95% CI 15%–62%) (*C. parvum*) of gorillas and 52% (95% CI 39%–65%) (adenovirus) and 13% (95% CI 4%–21%) (*C. parvum*) of chimpanzees. Both adenovirus and *C. parvum* had previously been detected in wild great ape populations and have received attention, given their zoonotic potential (*9*,*10*).

**Table 1:** Number of individual wild Cross River gorillas (*Gorilla gorilla diehli*) positive for enteric infection targets in Kagwene Gorilla Sanctuary and Mone River Forest Reserve, Cameroon (n = 18), 2011–2012

Assay target	Pathogen group	No. positive gorillas
*Cryptosporidium parvum*	Parasite	7
All adenovirus serotypes except 40 and 41	Virus	6
*Enterococcus faecalis*	Bacteria	5
Enterotoxigenic *Escherichia coli: E. coli* carrying virulence gene for heat-labile or heat-stable enterotoxin	Bacteria	1
Enteropathogenic *E. coli: E. coli* carrying gene (*eae*) encoding outer membrane protein intimin and causing pathogenesis through attachment/effacement of epithelial cells	Bacteria	1
*Escherichia coli* and *Shigella* species carrying invasion plasmid antigen H gene	Bacteria	1
*Salmonella bongori* and all subspecies of *Salmonella enterica*	Bacteria	1

**Table 2:** Number of individual wild eastern chimpanzees (*Pan troglodytes schweinfurthii*) positive for enteric infection targets in Gombe National Park, Tanzania (n = 56), 2016–2018

Assay target	Pathogen group	No. positive chimpanzees
All adenovirus serotypes except 40 and 41	Virus	29
Enterotoxigenic *E. coli: E. coli* carrying virulence gene for heat-labile or heat-stable enterotoxin	Bacteria	5
Enteroaggregative *E. coli: Escherichia coli* carrying a virulence gene (*aaiC*) associated with causing pathogenesis through aggregation in the intestinal mucosa	Bacteria	4
Enteropathogenic *E. coli: Escherichia coli* carrying gene (*bfpA*) encoding bundle-forming pilus and causing pathogenesis through attachment/effacement of epithelial cells	Bacteria	1
*Cryptosporidium parvum*	Parasite	7
All Enterovirus serotypes within *Enterovirus* genus	Virus	5
All *Giardia* species infecting humans	Parasite	5
*Trichuris trichiura* (*Trichocephalus trichiuris)*	Parasite	3
*Escherichia coli* and *Shigella* species carrying invasion plasmid antigen H gene	Bacteria	2
*Aeromonas hydrophila, caviae, veronii, jandaei, salmonicida, schubertii, popofii*	Bacteria	1
*Enterococcus faecalis*	Bacteria	1
Norovirus belonging to genogroup 2	Virus	1
Rotavirus A species from *Rotavirus* genus	Virus	1

## Conclusions

Real-time PCR testing of noninvasively collected wild gorilla and chimpanzee fecal samples from Cameroon and Tanzania provided evidence of widespread enteric infections and demonstrates their potential circulation in ape populations in Africa before 2018. Several pathogen targets detected in the ape species are highly relevant to humans, including those commonly associated with diarrheal disease, such as diarrheagenic *E. coli*, adenovirus, *Shigella* spp., *Giardia* spp., and enterovirus. As human–nonhuman primate contact increases in tropical forest communities, opportunities will continue to arise for both anthroponotic and zoonotic exchange and exposure (*11*).

Adenoviruses and *Cryptosporidium* species infect a broad range of hosts (including humans and nonhuman primates), can cause mild to severe disease, and are also associated with high rates of illness and death in children and immunocompromised persons, especially in developing countries (*12*). Although the pathogenesis of those organisms is less understood in nonhuman primate populations, they are of major zoonotic importance, given the increasing overlap between humans and wild primates and high HIV/AIDS prevalence in humans in regions inhabited by primate populations. Furthermore, because *C. parvum* and adenoviruses can spread through the fecal–oral route and persist in the environment for extended periods, diverse opportunities exist for direct and indirect transmission between humans and great apes (e.g., tourism and research activities, crop-raiding by apes, and events related to humans living in close proximity to parks).

Of note, many of the observed simian adenoviruses show high degrees of sequence relatedness to human strains, suggesting evidence of past cross-species transmission events and potential risk for such events in the future (*10*). Differentiating between strains was beyond the scope of this study, but the high detection rate of this viral target and its zoonotic potential warrants further characterization of this viral group and continued surveillance of great ape populations.

The first limitation of our study is that, because of logistical challenges and budgetary constraints, we were only able to focus our surveillance on 2 populations of great apes at specific points in time. In addition, sex and age classes sampled were representative of each ape population apart from infants, which are nearly impossible to sample noninvasively. 

Despite those challenges, our data provide insight into the diversity of enteric infections circulating in wild gorilla and chimpanzee populations before 2018. Detection of gene targets of zoonotic potential in 83% of gorillas and 70% of chimpanzees suggests potential health and disease transmission risks. These results are especially pertinent for monitoring these ape species given the previously documented cases of disease and epizootics (e.g., respiratory infections, polio, mange) in Gombe (*13*), and the lack of such in Cross River gorilla populations. As research, ecotourism, and forest encroachment in wild ape habitat increases, the risk for novel pathogen exposure is heightened, which could have catastrophic impacts on populations. Continued epidemiologic research among wild primate populations has the potential to predict which pathogens might enter both human and great ape populations as contact between species intensifies. Because pathogen exchange occurs across species boundaries, the potential for changes in pathogenicity and host specificity exists, which could have substantial adverse effects on human and wildlife health (*14*,*15*). 

Appendix More information about potentially zoonotic enteric infections in gorillas and chimpanzees, Cameroon and Tanzania.
