# Supplementary material for: Potentially Zoonotic Enteric Infections in Gorillas and Chimpanzees, Cameroon and Tanzania
Source: Emerg Infect Dis. 2024 Mar;30(3):577–80. doi: 10.3201/eid3003.230318 (PMC10902540; doi:10.3201/eid3003.230318)
Supplement: Appendix — More information about potentially zoonotic enteric infections in gorillas and chimpanzees, Cameroon and Tanzania. [file 23-0318-Techapp-s1.pdf]

Article DOI: https://doi.org/10.3201/eid3003.230318

EID cannot ensure accessibility for supplementary materials supplied by authors. Readers who have difficulty accessing supplementary content should contact the authors for assistance.

Potentially Zoonotic Enteric Infections in Gorillas and Chimpanzees, Cameroon and Tanzania

Appendix

Appendix Table. The 39 unique enteric pathogen targets and control targets employed to screen wild Cameroonian Cross River gorillas (*Gorilla gorilla diehli*) and Tanzanian eastern chimpanzees (*Pan troglodytes schweinfurthii*) fecal samples via the TaqMan Array Card.

Code	Assay	Gene Target
NOR1_1	Norovirus GI	*ORF 1–2*
NOR2_1	Norovirus GII	*ORF 1–2*
CLDA_1	*C. difficile* tcdA	*tcdA*
CLDB_1	*C. difficile* tcdB	*tcdB*
SALS_1	*Salmonella* spp.	*ttrRSBCA*
CLDP_1	*C. difficile* paLOC	*non-coding IS*
ENHI_1	*Entamoeba histolytica*	*18S*
GIAR_1	*Giardia* spp	*18S*
AERO_1	*Aeromonas* spp.	*glycosyltransferase*
ENFM_1	*Enterococcus facium*	*ddl*
EIES_1	*Escherichia coli/Shigella* (ipaH gene)	*ipaH*
EPE1_1	*Escherichia coli* (eae gene)	*eae*
EPE2_1	*Escherichia coli* (bfpA gene)	*bfpA*
EAE1_1	*Escherichia coli* (aaiC gene)	*aaiC*
EAE2_1	*Escherichia coli* (aatA gene)	*aatA*
ETLT_1	*Escherichia coli* (heat-labile enterotoxin)	*LT*
ETST_1	*Escherichia coli* (heat-stable enterotoxin)	*STh/STp*
STX1_1	Shiga toxin/Shiga-like toxin 1	*stx1*
STX2_1	Shiga-like toxin 2	*stx2*
AD4X_1	Adenovirus 40/41	*fiber protein*
ENTV_4	Enterovirus	*polyprotein*
ADEV_1	Adenovirus	*hexon*
ASTR_1	Astrovirus	*capsid*
ENFC_1	*Enterococcus faecalis*	*ddl*
CACO_1	*Campylobacter coli*	*cdtB*
CAJE_1	*Campylobacter jejuni*	*hipO*
ROTA_1	Rotavirus A	*NSP3*
ROTB_1	Rotavirus B	*NSP2*
ROTC_1	Rotavirus C	*VP6*
ROTN_1	Rotavirus non-typable	*NSP2*
YERS_1	*Yersinia* spp.	*pal*
MYTB_1	*Mycobacterium tuberculosis*	*IS6110*
VICH_1	*Vibrio cholerae*	*ompW*
VTOX_1	*Vibrio cholerae* toxigenic	*ctxA*
SAPX_1	Sapovirus I/II/IV	*RdRp*
SAPV_1	Sapovirus V	*RdRp*
CRPA_1	*Cryptosporidium parvum*	*hypothetical protein*
ASLU_1	*Ascaris lumbricoides*	*ITS*
TRTR_1	*Trichuris trichuria*	*18S*
MS2_1	MS2 phage for extraction control	*MS2g1*
RNP3_1	Human nucleic acid control (Rnase P)	*RnaseP*
